# Wetland seed dispersal by white-tailed deer in a large freshwater wetland complex

**DOI:** 10.1093/aobpla/plx074

**Published:** 2017-12-23

**Authors:** Kelley L Flaherty, James S Rentch, James T Anderson

**Affiliations:** 1 College of Science, Technology and Math, Alderson Broaddus University, Philippi, WV, USA; 2 Division of Forestry and Natural Resources, West Virginia University, Morgantown, WV, USA

**Keywords:** Appalachians, endozoochory, *Juncus effusus*, *Odocoileus virginianus*, *Oxalis dillenii*, West Virginia, wetland plants

## Abstract

Mechanisms of long-distance dispersal are important in establishing and maintaining plant populations in isolated wetland habitats. White-tailed deer (*Odocoileus virginianus*) have been cited as long-distance dispersers of both native and exotic plant species in North America; however, knowledge regarding their influence in wetlands is limited. Given traditional classification methods for seed dispersal, white-tailed deer are not likely viewed as important dispersal mechanism for wetland plants. We collected naturally deposited white-tailed deer faecal pellet piles from wetlands in Canaan Valley, West Virginia, USA. Pellet piles were cold-stratified and germinated seedlings over a layer of sterile potting mix. The percentage of germinated seedlings with a facultative wetland (FACW) or obligate wetland (OBL) plant indicator status were compared to the frequency of occurrence to those of germinated plants with facultative upland (FACU) or upland (UPL) indicator status. We identified 38 species. Of these, 1 % were UPL, 38 % were FACU, 18 % were FACW and 21 % were OBL. Graminoid species accounted for 42 %; forbs and woody species accounted for 29 % each. Our research has suggested that endozoochory by herbivores contributes to long-distance dispersal of wetland plants.

## Teaser

Understanding the dispersal of wetland plants is important to the conservation of species in patchy or isolated wetland environments. We explored the potential for wetland seed dispersal by white-tailed deer (Odocoileus virginianus) in a high-elevation wetland complex in Canaan Valley, West Virginia by collecting and germinating deer feces in a controlled environment. Our results suggest that seed dispersal by large herbivores may be a more prevalent mechanism for wetland plants than previously recognized. This mechanism allows for the potential for long-distance dispersal over upland habitat from isolated wetland environments.

## Introduction

Much of the research concerning the effects of deer on plant communities have focused on their role as browsers and the potential for overbrowsing (Alverson and Waller 1997; Stromayer and Warren 1997; Royo *et al.* 2010). However, several studies have looked at the role of white-tailed deer as both seed predators and seed dispersers (Cambell and Gibson 2001; Vellend *et al.* 2003; Furedi and McGraw 2004; Myers *et al.* 2004; Bartuszevige and Endress 2008). Due to their large home-range size (e.g. 66–235 ha in WV; Campbell *et al.* 2004) and the potential to retain material in the digestive tract for three or more days (Mautz and Petrides 1971; Moussie *et al.* 2005), white-tailed deer have the potential to carry seeds great distances (Janzen 1984). Vellend *et al.* (2003) suggested that this would result in 95 % of germinable seeds being deposited >100 m from the parent plant and 30 % deposited >1 km from the parent plant. This may have a considerable effect on metapopulation initiation, growth and gene flow of irregularly distributed and rare plant species (Myers *et al.* 2004).

Myers *et al.* (2004) found that 72 species of forest and old-field plants germinated from faecal pellet samples with a mean of 38 germinations per pellet group. The germinated seeds ranged from forbs to tree species. In contrast, Cambell and Gibson (2001) found only two species germinated from 22 pellet group samples. Although deer may play an important role in the dispersal of seed for many plants, they may also play a role in the spread of exotic species (Cambell and Gibson 2001; Bartuszevige and Endress 2008; Williams *et al*. 2008).

Traditional methods of determining dispersal mechanism are to classify plants based on seed characteristics. However, seeds of many species do not have special characteristics and some may have more than one dispersal method (Drezner *et al.* 2001). Also, though a seed may be physically adapted for one type of dispersal, it is not known whether or not that seed could still germinate if consumed by an herbivore. Drezner *et al.* (2001) discussed the methods of seed dispersal of riparian plants and argued that obligate wetland plants are more often dispersed by water or wind while upland plants are more often dispersed by animals. This study evaluated few obligate or facultative wetland plants (*n* = 10) and many upland or facultative upland plants (*n* = 46).

Obligate or facultative wetland plants with highest fitness would disperse via methods that would ensure that seeds are deposited in favourable growing conditions. Thus, many wetland plants may not be adapted to long-distance seed dispersal by large ungulate herbivores such as white-tailed deer and are thus consumed rather than carried by deer. We examined the potential for white-tailed deer to disperse wetland species by collecting and germinating seeds from natural faecal pellet piles from the Canaan Valley, West Virginia, USA high-elevation wetland complex to determine the species of germinable seeds they contain as well as what percentage come from obligate and facultative wetland plant species.

## Methods

### Study area

Canaan Valley, located in Tucker County, West Virginia, USA is the highest elevation valley east of the Rocky Mountains (Fortney 1993). As such, the climate and, accordingly, the vegetation of the valley are more similar to northern boreal forests than to the deciduous forests of surrounding West Virginia. Once home to large stands of red spruce (*Picea rubens*), intense logging followed by fires drastically changed the soils and vegetation of the valley to their present condition (Fortney 1993). The valley floor averages 975 m above sea level. This, coupled with surrounding mountains which rise 150–240 m above the valley, creates a relatively cool, moist climate and a short growing season of ~90 days (Beverage 1967). The average annual precipitation is 137 cm and annual snowfall is 305 cm (Fortney 1993). These characteristics set this area apart from low-elevation wetland and upland areas in the surrounding counties.

Canaan Valley encompasses ~176 km^2^ (17600 ha) of land. Approximately 20 % of the land area is made up of various wetland community types and another 23 % is northern hardwood forest (Flaherty 2014). Of all the high-elevation wetlands in West Virginia, Canaan Valley is home to the largest contiguous wetland complex (3000 ha; Byers *et al.* 2007). This wetland complex is home to 27 distinct wetland community types ranging from quaking aspen (*Populus tremuloides*) groves to sphagnum (*Sphagnum* spp.) and polytrichum (*Polytrichum* spp.) bogs and includes many rare wetland plant communities (Fortney *et al.* 2015).

Canaan Valley is home to Canaan Valley State Park (2433 ha) and the Canaan Valley National Wildlife Refuge (6729 ha) and, at the time of this study, land owned by the Canaan Valley Institute (1298 ha), a non-profit organization. Sampling took place on a combination of these three public access properties in the valley.

### Field methods

We collected fresh white-tailed deer faecal pellet groups twice monthly from May to December 2005 and 2006 in wetland habitats. In 2005, we collected pellet piles from along three 300 m transects through both herbaceous, shrub and forested wetlands within the Canaan Valley National Wildlife Refuge and Canaan Valley State Park. In 2006, six additional wetland transects were added for a total of nine transects at sampling locations within the refuge and state park (Flaherty 2014). We located transect near known deer tails to increase the opportunities to collect fresh samples. We collected piles by walking along transects and selecting fresh pellet piles from those within view. We chose piles that were visibly moist and dark in colour. All visible pellets were removed from surrounding vegetation and placed in plastic bags. We did not observe any obvious fallen seeds on pellet piles. We attempted to collect pellets without including surrounding debris, including seeds that may have come from the surrounding vegetation rather than through endozoochory. Samples were then stored in sealed plastic bags and refrigerated at 4 °C to retard germination and fungal growth.

Individual pellets were broken by rinsing gently through a 0.5-mm sieve. The resulting seeds and residual matter were stored in Petri dishes at 4 °C for a period of 3 months to simulate overwintering (Myers *et al.* 2004). In March, the seeds were spread on top of a layer of sterile growing medium in 10 cm diameter planting pots and kept moist and were subsequently housed in a greenhouse and watered when needed. The pots were kept in greenhouse conditions until germination and identification of species germinated was possible. The number of each plant species that germinated from each pellet group was recorded.

### Statistical analysis

We determined the wetland indicator status of all plants germinated from pellet piles. For the purposes of our analysis, we pooled the species that had OBL (obligate wetland: plants with >99 % frequency of occurrence in wetlands) or FACW (facultative wetland: plants with 67–99 % frequency of occurrence in wetlands; Lichvar *et al.* 2014) status as these plants are most likely to be adapted for growth and dispersal in wetlands. We used wetland indicator status values for Eastern Mountains and Piedmont region as listed in the U.S. Army Corps of Engineers National Wetland Plant List (Lichvar *et al.* 2014). We excluded species that were considered facultative (facultative: FAC; plants that are equally likely to be found in uplands and wetlands), species with an unknown status and species that were only identified to the genus level. We also classified each species as graminoid, forb or woody species (including trees and shrubs). We used a chi-square test to compare the proportion of species germinated that were either UPL (upland: plants with <1 % frequency of occurrence in wetlands) or FACU (facultative upland: plants with 1–33 % frequency of occurrence in wetlands) with those that were either FACW or OBL. We considered differences at the *P* < 0.05 level to be significant. We defined the frequency of occurrence as the number of pellet piles in which each species occurred out of the number of total germinated pellet piles. We defined abundance as the total number of seedlings present in all samples. We also compared the frequency of germination events that were either UPL or FACU with those that were either FACW or OBL. We repeated these tests for the proportion and frequency of germination of plants that were graminoid, forbs or woody. Finally, we compared the frequency of native and exotic plants germinating in the pellet piles.

## Results

We collected 55 pellet piles in 2005 and 160 pellet piles in 2006. Of those collected in 2005, 45 % of the pellet piles planted germinated at least one species resulting in a total of 14 species (Table 1). Of those collected in 2006, 38 % of the pellet piles germinated at least one species resulting in 31 species germinating (Table 1). Thirty-eight species were identified over a period of 2 years (two additional seedlings were identified to the genus level).

**Table 1.** Species germinated from 55 white-tailed deer pellet piles collected in 2005 from three locations and 160 white-tailed deer pellet piles collected in 2006 from nine locations in Canaan Valley, West Virginia, USA. Binomial nomenclature accessed from the Integrated Taxonomic Information System at http://www.itis.gov. ^a^An * indicates plants are exotic to the USA. ^b^Forms are designated by G = graminoid; F = forb; and W = woody species. ^c^Life forms are designated by A = annual and P = perennial. ^d^The wetland indicator status of each plant is described by OBL = obligate wetland, FACW = facultative wetland, FAC = facultative, FACU = facultative upland, and UPL = upland.

Latin name^a^	Common name	Form^b^	Life^c^	Frequency		Abundance		Indicator status^d^
				2005	2006	2005	2006	
*Agrostis perennans*	Upland bentgrass	G	P		2		13	FACU
Anthoxanthum odoratum*	Sweet vernal grass	G	P		3		4	FACU
*Apocynum cannabinum*	Indian hemp	F	P		1		2	FACU
*Carex crinita*	Fringed sedge	G	P	3	3	10	8	OBL
*Carex debilis*	White-edge sedge	G	P		4		7	FAC
*Carex folliculata*	Northern long sedge	G	P		2		4	OBL
*Carex leptalea*	Bristly-stalked sedge	G	P		1		1	OBL
*Crataegus* spp.	Hawthorne	W	P		1		1	FAC
*Danthonia compressa*	Flattened oatgrass	G	P	2	4	12	17	FACU
*Dichanthelium clandestinum*	Deertongue grass	G	P		3		12	FAC
Elaeagnus umbellata*	Autumn olive	W	P		1		2	FAC
*Festuca subverticillata*	Nodding fescue	G	P		1		1	FACU
*Glyceria canadensis*	Rattlesnake mannagrass	G	P	1	1	1	1	OBL
*Glyceria striata*	Fowl mannagrass	G	P		1		1	OBL
Holcus lanatus*	Velvet grass	G	P		2		3	FAC
*Hypericum densiflorum*	Glade St. John’s-wort	W	P		1		2	FACW
*Juncus canadensis* Laharpe	Canadian rush	G	P	1		2		OBL
*Juncus effusus*	Common rush	G	P	4	4	5	7	FACW
*Lobelia inflata*	Indian tobacco	F	A	2	1	2	1	FACU
*Lycopus uniflorus*	Northern bugleweed	F	P	1		1		OBL
*Mimulus ringens*	Allegheny monkey flower	F	P	1		9		OBL
*Muhlenbergia schreberi*	Nimblewill	G	P	1		1		FAC
*Oxalis dillenii*	Wood sorrel	F	P	6	11	18	34	FACU
*Plantago rugelii*	Blackseed plantain	F	P	2		1		FACU
*Poa* spp.	Bluegrass spp.	G	P	1		3		FACW–FACU
*Persicaria pensylvanicum*	Pinkweed	F	A		3		4	FACW
*Prunus serotina*	Black cherry	W	P		1		1	FACU
*Ranunculus acris*	Tall buttercup	F	P		1		2	FAC
Rosa multiflora*	Multiflora rose	W	P		2		3	FACU
Rumex acetosella*	Sheep sorrel	F	P		1		3	UPL
*Spiraea alba*	Meadowsweet	W	P	1	2	1	4	FACW
*Spiraea tomentosa*	Steeplebush	W	P	2	1	3	1	FACW
Trifolium repens*	White clover	F	P		5		20	FACU
*Vaccinium myrtilloides*	Velvetleaf blueberry	W	P		2		3	FACW
Veronica officinalis*	Common speedwell	F	P	1		2		FACU
*Viburnum nudum*	Wild raisin	W	P		1		1	OBL
*Viburnum recognitum*	Smooth arrowwood	W	P		1		1	FAC
*Viola sagittata*	Arrowleaf violet	W	P		1		2	FAC

We found no significant difference between the proportion of species germinated that have a FACW (*n* = 8) or OBL (*n* = 9) wetland indicator status (40 %) and those that had a FACU (*n* = 12, 35 %) or UPL (*n* = 1) status (35 %, *P* > 0.05; Table 2). There was also no difference in the combined frequency of FACW and OBL (38 %) plants versus FACU plants and UPL (*n* = 1) plants (35 %, *P* > 0.05; Table 2). The combined abundance of seedlings that were FACU or UPL (57 %; Table 2) was significantly more than the combined proportion of FACW (13 %) and OBL (17 %) stems counted (χ^2^ = 22.222, df = 1, *P* < 0.001; Table 2).

**Table 2.** The number of species, frequency and abundance of species germinating from white-tailed deer pellet piles collected in Canaan Valley, West Virginia, USA during 2005 and 2006 separated by wetland status and growth form. **Significantly higher at *P* < 0.01. ***Significantly higher at *P* < 0.001.

	Number of species		Frequency		Abundance	
	*n*	%	*n*	%	*n*	%
Wetland status						
OBL and FACW	17	40	36	38	69	30
UPL and FACU	13	35	45	47	134***	57
Growth form						
Forb	11	29	36**	37	99***	42
Graminoid	16	42	44**	45	113***	48
Woody	11	29	17	18	25	10
Native status						
Native	30	81	97***	87	202***	85
Exotic	7	19	15	13	35	15

Both the proportion of forb species (*n* = 11, 29 %) and woody species (*n* = 11, 29 %) that germinated from pellet piles were less than the proportion of graminoid species (*n* = 16, 42 %) that germinated; however, the difference was not significant (*P* > 0.05). The frequency of both graminoids (*n* = 44, 45 %) and forbs (*n* = 36, 37 %) was significantly higher than for woody species (*n* = 17, 18 %, χ^2^ = 11.9, df = 2, *P* < 0.01; Table 2). The abundance of individual seedlings that were forbs (42 %) and graminoids (48 %) were significantly greater than the abundance of woody seedlings (10 %, χ^2^ = 56.1, df = 2, *P* < 0.001; Table 2), but the abundance of graminoids and forbs did not significantly differ (*P* > 0.05).

Lastly, we found the frequency of exotic seedlings (*n* = 15, 13 %) was significantly lower than the frequency of native seedlings germinating (*n* = 97, 87 %, χ^2^ = 60.03, df = 1, *P* < 0.001; Table 2). The abundance of exotic seedlings (*n* = 35, 15 %) was significantly lower than the frequency of native seedlings germinating (*n* = 202, 85 %, χ^2^ = 117.68, df = 1, *P* < 0.001; Table 2).

## Discussion

### Seedling germination

We observed no difference in the proportion of wetland and upland plant species germinating in pellet piles collected. However, there were a significantly higher abundance of UPL and FACU seedlings germinating. While a difference in frequency of plants seems to agree with the hypothesis that upland plants may be more prone to dispersal by white-tailed deer than wetland plants, there was no difference in the proportion of wetland and upland species. The difference in frequency could be attributed to the successful germination of several FACU plants, such as *Oxalis dillenii*, which occurred in 11 pellet piles collected in 2006.

Although we did observe the presence of FACW and OBL wetland species germinating in pellet piles, these observations may underestimate the actual presence of germinable wetland species in pellet piles. All pellet piles were grown in moistened but not saturated soil. Alternate conditions may be required to germinate all wetland species present in a sample. Saturated conditions would be present for at least some of the pellet piles deposited naturally by white-tailed deer. Future studies should simulate these saturated conditions.

### Seed dispersal

Our study shows that some wetland plants can be successfully dispersed by white-tailed deer. As they have relatively large home ranges compared to other mammal species (Willson 1993), deer have the potential to disperse seeds of wetland species between isolated wetland patches. As dispersers, white-tailed deer could play a role in maintaining or enhancing metapopulations of wetland plants patchily distributed in an upland matrix (Vellend *et al.* 2003). Maintenance of metapopulations may also enhance overall plant species diversity within a wetland complex. However, white-tailed deer may also impact wetland restoration by successfully dispersing seeds of some exotic species (Vellend 2002; Myers *et al.* 2004; Bartuszevige and Endress 2008). We found seven exotic species germinated in our pellet piles, with the most abundant species being sweet vernal grass (*Anthoxanthum odoratum*).

A high proportion of germinated species were graminoids. Although there was no significant difference between number of species that were graminoids, forbs or woody species, there was a significantly higher frequency and abundance of graminoid seedlings than woody seedlings. Endozoochory by large mammals is often associated with the co-evolution of fleshy fruits (Willson 1993; Beck and Vander Wall 2010); however, Janzen (1984) suggested that the foliage of forbs and grasses may attract herbivores to disperse small seeds. While fleshy fruit may attract seed dispersers, the mastication process may destroy larger seeds. Small, rounded, hard seeds may support passage through the digestive tract of ungulates better than larger seeds (Moussie *et al.* 2005; Iravani *et al.* 2011). Our results support this ‘foliage as fruit’ hypothesis (Janzen 1984) and suggest that graminoids may be more likely to be dispersed by herbivores that their traditional methods of classification suggest.

Successful dispersal and subsequent establishment of graminoid species by deer may help to maintain or expand herbaceous openings at both wetland and upland sites. Large areas of Canaan Valley consist of wet meadow habitat. White-tailed deer may help to maintain these meadows, not only by reducing the growth of woody species through browse, but also by dispersing the seeds of graminoid species (Bartuszevige and Endress 2008; Iravani *et al.* 2011). Many studies have examined the influence of white-tailed deer on upland forest habitat. They are thought to affect the regeneration of forest species through the overbrowsing of seedlings (Rooney 2009; Griscom *et al.* 2011; Tanentzap *et al.* 2012) but may also alter the ground cover through trampling, soil exposure or disturbance of leaf litter (Knight *et al.* 2009). As dispersers of graminoid species, deer also may reduce the capacity for woody species regeneration by increasing the prevalence of grass species within the ground cover in both upland and wetland environments.

## Conclusions

A key question raised by the results of this study is to what degree these data are representative of the species present in the Canaan Valley ecosystem that are germinable when passed through the digestive system of white-tailed deer and what percentage of the total seeds consumed remain intact for each species. Of those species recorded in Canaan Valley, 20 % are obligate wetland species (OBL), 18 % are facultative wetland species (FACW), 15 % are facultative species (FAC), 24 % are facultative upland species (FACU) and only 3 % are upland species (UPL). We believe the abundance and frequency of seeds in pellet piles are likely not a good indicator of the proportions of these plants in the diet of white-tailed deer and should not be used as an indicator of such. Additionally, of those plants eaten, some may not have germinated under the conditions provided. We recorded the presence of species at our study sites that have been reported by others as having germinated from deer pellet piles but did not occur in our samples (e.g. *Achillea millefolium* and *Festuca ovina*; Myers *et al.* 2004; Iravani *et al.* 2011). In the future, we suggest the division of samples to allow the application of different treatments that could promote the germination of different species. All studies that we are aware of concerning the germination of plant species from pellet piles have been conducted under highly controlled conditions. It remains to be seen, however, if these results translate to pellet piles that germinate under more natural conditions. Although, we sampled only from wetland sites, herbivores may carry wetland seeds to upland habitat not suitable for germination. Future studies should examine the potential for seed loss through dispersal to unsuitable environments.

## Sources of Funding

Funding for this project was provided by Canaan Valley Institute, Regional Research Institute, and West Virginia University Davis College of Agriculture, Natural Resources and Design (McIntire–Stennis) (grant number WVA00050). J.T.A. was supported by the National Science Foundation under Cooperative Agreement (OIA-1458952) during manuscript preparation.

## Contributions by the Authors

K.L.F. conceived of and conducted the experiment with input from J.T.A. and J.S.R. Seedlings were identified by K.L.F. with assistance from W. Grafton. K.L.F. wrote the manuscript with input from J.T.A. and J.S.R.

## Conflict of Interest

None declared.
